# Incorporating the Number of PLN into the AJCC Stage Could Better Predict the Survival for Patients with NSCLC: A Large Population-Based Study

**DOI:** 10.1155/2020/1087237

**Published:** 2020-12-11

**Authors:** Xiaoling Shang, Zhenxiang Li, Jiamao Lin, Haining Yu, Chenglong Zhao, Haiyong Wang, Jian Sun

**Affiliations:** ^1^Shandong Cancer Hospital and Institute, Cheeloo College of Medicine, Shandong University, Jinan, Shandong 250012, China; ^2^Department of Clinical Laboratory, Shandong Cancer Hospital and Institute, Shandong First Medical University and Shandong Academy of Medical Sciences, Shandong University, Jinan 250117, China; ^3^Department of Radiation Oncology, Shandong Cancer Hospital and Institute, Shandong First Medical University and Shandong Academy of Medical Sciences, Jinan 250117, China; ^4^Department of Internal Medicine-Oncology, Shandong Cancer Hospital and Institute, Shandong First Medical University and Shandong Academy of Medical Sciences, Jinan 250117, China; ^5^Personnel Division, Shandong Cancer Hospital and Institute, Shandong First Medical University and Shandong Academy of Medical Sciences, Jinan 250117, China; ^6^Department of Pathology, Shandong Cancer Hospital and Institute, Shandong First Medical University and Shandong Academy of Medical Sciences, Jinan 250117, China; ^7^Department of Thoracic Surgery, Shandong Cancer Hospital and Institute, Shandong First Medical University and Shandong Academy of Medical Sciences, Jinan 250117, China

## Abstract

**Purpose:**

This study aimed to investigate the application of the number of positive lymph nodes (PLNs) in tumor, node, metastasis (TNM) staging system of non-small cell lung cancer (NSCLC) patients. *Patients and Methods*. We screened a total of 15820 patients with resected NSCLC between 2004 and 2015 from SEER database. The X-tile model was used to determine the cutoff values of the number of PLNs. Overall survival (OS) curves were plotted using the Kaplan–Meier method, and the differences among the individual groups were defined using the log-rank test. Cox regression model was used to perform univariate and multivariate analyses and to assess the association between the number of PLNs and OS.

**Results:**

In this study, using the X-tile model, we screened three different cutoff values, including nN0, nN1–3, and nN4-. Survival curves demonstrated that our defined nN stage had a significant predictive value for OS (*P* < 0.001). In the univariate and multivariate Cox analyses, the result showed that nN stage was a significant prognostic factor of OS for NSCLC patients (*P* < 0.001). Subsequently, we classified the patients into five subgroups based on the combination of pN and nN stages, including pN0 + nN0, pN1 + nN1-3, pN2 + nN1-3, pN1 + nN4-, and pN2 + nN4-. Moreover, survival curves revealed significant differences among these five groups (*P* < 0.001).

**Conclusion:**

A combination of pathological LNs (pN) and the number of LN (nN) involvement in NSCLC patients had a better prognostic value than the current TNM staging system based on only pN stage.

## 1. Introduction

Lung cancer is considered a severe disease worldwide and is the leading cause of cancer death, where 85% comprises non-small cell lung cancer (NSCLC) [1, 2]. According to the 2018 Annual Report of Chinese National Cancer Centre, lung cancer had the highest incidence and mortality rates among all cancers. Thus, accurate staging, proper treatment, and better prognosis are essential to improve the survival of NSCLC patients [3].

The tumor, node, metastasis (TNM) staging system, based on primary tumor character (T), nodal involvement (N), and distant metastasis (M), has a significant impact on therapeutic options and prognosis, which is essential in determining ways to deliver care to patients. There were some updates of T and M classification in the eighth version, but the N classification remained unchanged [4, 5]. Since pathologically positive lymph node (PLN) indicates a higher recurrence risk, accurate assessment of LN is essential in creating treatment strategy and prognostic care. Even lymphadenectomy is recommended to be performed in lung resection surgery to ensure a significant survival, but still, 25% to 50% of early-stage lung cancer patients have a disease recurrence, suggesting that the current staging system is still inaccurately sufficient to assess NSCLC.

PLN was also considered a strong prognostic factor in NSCLC. It has been proven that the number of PLNs has a fundamental prognostic value in gastric, colorectal, breast, bladder, and esophageal cancer [6]. Furthermore, the number of PLNs was recommended as a staging parameter in these cancers by the National Comprehensive Cancer Network guidelines, but it was not systematically illustrated in NSCLC. Only LN stations were elaborated in the latest 8th edition of the TNM staging system, with no more details about the exact number in the classification.

Hence, we used the Surveillance, Epidemiology, and End Results (SEER) database, selected 15,820 NSCLC patients who underwent complete systematic resection of the LNs, and retrospectively investigated the survival rate affected by the PLN number. The X-tile model was used to determine the threshold of LN number. Comprehensively, we attempted to combine the PLN number and the current TNM staging system to provide a more precise treatment guideline and establish a better prognosis.

## 2. Patients and Methods

### 2.1. Data Source

The SEER database provided a comprehensive source of cancer statistics, including detailed information on patient demographics, pathologic diagnosis, treatment strategy, and prognosis. Using this database, we selected and analyzed a total of 15,820 patients between 2004 and 2015 using the SEER *∗* Stat 8.3.5 software. All the patients had NSCLC without distant metastasis and underwent lung resection surgery involving the dissection of hilar and mediastinal LNs. The number of harvested LNs was at least 10, which would ensure an optimal prognosis and a more accurate staging. Staging was classified according to the 6th edition of the TNM staging system. The patients with incomplete information were all excluded.

### 2.2. Ethical Statement

This study was performed according to the Declaration of Helsinki. Permission was granted in accessing the SEER database. The Ethics Committee of Shandong Cancer Hospital and Institute approved this study. Personal information was not included. All the data were obtained from the public database which did not involve personal information, so the informed consent was not required.

### 2.3. Statistical Analyses

The X-tile model was used to determine the cutoff values of the number of PLNs. Survival curves were plotted using the Kaplan–Meier method, and differences among the individual groups were defined using the log-rank test. Cox regression model was used to perform univariate and multivariate analyses and to assess the association between the number of PLNs and OS. All statistical methods were two-sided, and *P* < 0.05 was considered to be statistically significant. Statistical Package for the Social Sciences 22.0 software was used in data analysis.

## 3. Results

### 3.1. Patients' Characteristics

A total of 15,820 NSCLC patients between 2004 and 2015 were selected from the SEER database for analysis as listed in Table 1. Among these, 38.9% were younger than 65 years old, and 61.1% were older than 65 years old. Regarding race, 85.2% were White, 7.8% were Black, and 6.9% were from other races. Female patients had a percentage of 47.5%, while male patients had 52.5%. Additionally, a major group of patients belonged to adenocarcinoma (60.6%) and the others to squamous (39.4%). The percentages of patients in T1, T2, T3, and T4 stages were 35.2%, 50%, 6.6%, and 8.2%, respectively. A majority of patients were in stage pN0 (63.5%), and the percentage of patients in stages pN1, pN2, and pN3 were 20.4%, 15.8%, and 0.3%, respectively. A total of 65.1% of patients belonged to the nN0 stage, and 23.2% and 11.7% patients were in stage nN1-3 and stage nN4, respectively.

### 3.2. Determination of Cutoff Values for PLNs

**Figure 1:**
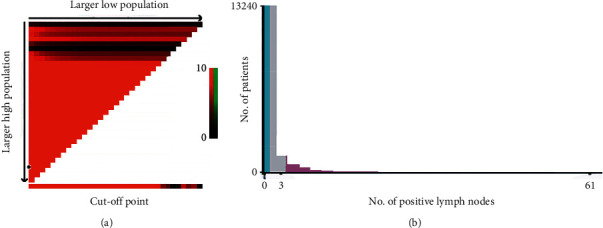
The optimal threshold of the number of positive lymph nodes determined by the X-tile model. (a) X-tile plots based on no. of positive lymph nodes. (b) The optimal cutoff point is shown by the blue (no. of PLN = 0), gray (1 ≤ no. of PLN ≤ 3), and violet panel (no. of PLN ≥ 4).

The cutoff values of PLNs were determined using the X-tile model. Survival curves were analyzed using the Kaplan–Meier method, and log-rank test was used to compare the differences. According to LN numbers, the groups were divided into low (*n* = 0), medium (1 ≤ *n* ≤ 3), and high (3 < *n* ≤ 61) using the X-tile model (Figures 1(a) and 1(b)). Therefore, based on the threshold, three stages were determined as nN0, nN1-3, and nN4-.

### 3.3. Survival Analysis

**Figure 2:**
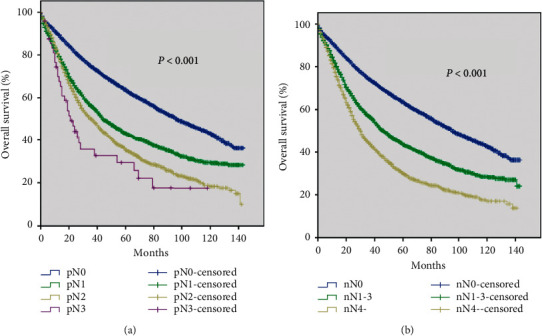
Survival curves of NSCLC patients based on the (a) current pN stage (*P* < 0.001) and (b) hypothesized nN stage (*P* < 0.001).

**Figure 3:**
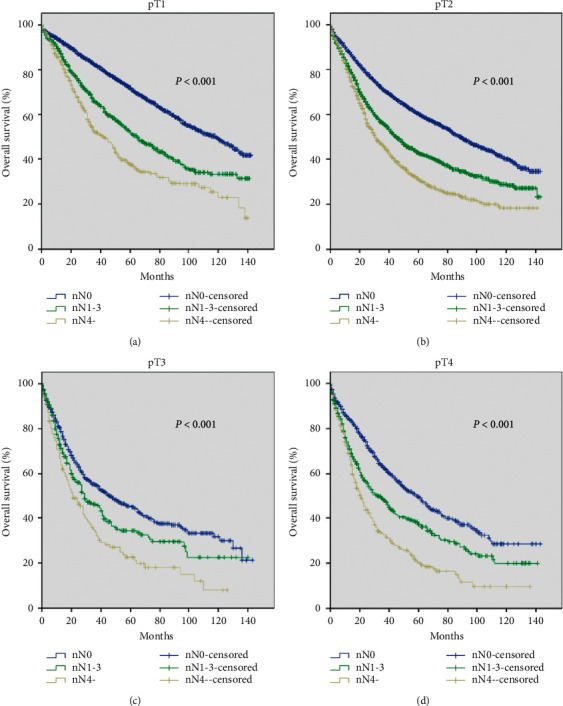
Survival curves of different nN stages based on different T stages. Survival curves of different nN stages based on (a) pT1 stage (*P* < 0.001), (b) pT2 stage (*P* < 0.001), (c) pT3 stage (*P* < 0.001), and (d) pT4 stage (*P* < 0.001).

By evaluating the influence of PLN number on NSCLC patients, we first analyzed the OS according to the pN stage. The pN stage was widely accepted as a prognostic factor, and our data showed a significant difference among the pN groups (*P* < 0.001) (Figure 2(a)). Using the same method, we found that the OS curve was well distinguished by the defined nN stage (*P* < 0.001) (Figure 2(b)). These data suggested that our defined nN stage had a significant predictive value for prognosis. Subsequently, patients with different pathological tumor (pT) classifications were grouped according to nN status. We plotted survival curves of nN status based on different pT stages, including pT1, pT2, pT3, and pT4. In Figure 3(a), we found that the survival curves of stages pT1nN0, pT1nN1-3, and pT1nN4- were obviously separated and significantly distinguished (*P* < 0.001). Similar results of the survival curves of stages nN0, nN1, and nN4- were observed in the pT2, pT3, and pT4 groups (all *P* < 0.001) (Figures 3(b)–3(d)).

### 3.4. Survival on the pN and the nN Stages

In Table 2, Cox proportional hazards model was used to evaluate the prognostic value of the baseline characteristics. Univariate and multivariate analysis revealed that variables including age, race, sex, histology, pT stage, and pN stage were all significant prognostic factors on OS for NSCLC patients (all *P* < 0.001). In multivariate analysis, the pN stage was an independent prognostic factor for survival (pN1 vs. pN0: hazard ratio (HR), 1.630; 95% confidence interval (CI), 1.534–1.733; *P* < 0.001; pN2 vs. pN0: HR, 2.157; 95% CI, 2.023–2.300; *P* < 0.001; and pN3 vs. pN0: HR, 2.799; 95% CI, 1.983–3.949; *P* < 0.001).

To further clarify the significance of nN stage, we then compared different factors including nN stage on OS in NSCLC patients. Univariate and multivariate analysis revealed that all the factors, including age, race, sex, histology, pT stage, and nN stage, were considered to have independent prognostic values (all, *P* < 0.001). In multivariate analysis, the nN stage was an independent prognostic factor of OS (nN1–3 stage vs. nN0 stage: HR, 1.657; 95% CI, 1.564–1.756; *P* < 0.001 and nN4- stage vs. nN0 stage: HR, 2.371; 95% CI, 2.213–2.540; *P* < 0.001). Factors that affected the OS using univariate and multivariate analyses are listed in Table 3.

### 3.5. Survival Curves Based on the Combination of pN and nN Stages

**Figure 4:**
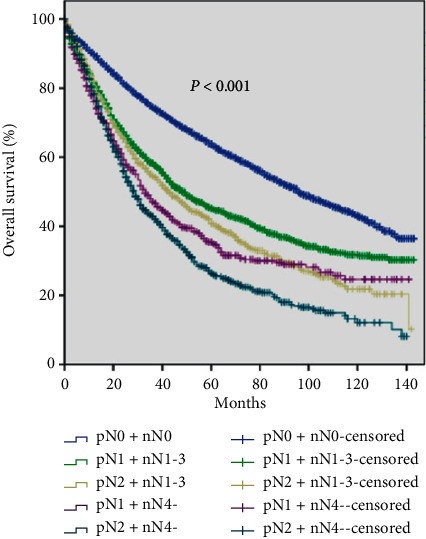
Survival curves of patients with stages pN0 + nN0, pN1 + nN1-3, pN2 + nN1-3, pN1 + nN4-, and pN2 + nN4-, respectively (*P* < 0.001).

Nodal status is an important factor for TNM staging system. The pN stage has already exhibited an essential prognostic value and was involved in the classification, but little is known about the value of nN as a complementary for classification. We classified the patients into five subgroups based on the combination of the pN and nN stages, namely, pN0 + nN0, pN1 + nN1-3, pN2 + nN1-3, pN1 + nN4-, and pN2 + nN4-. Subsequently, we drew survival curves of these subgroups. The result revealed that NSCLC patients among these five groups had significantly different OS (log-rank test, *P* < 0.001) (Figure 4).

## 4. Discussion

The lung cancer TNM staging system is developed based on sophisticated statistical analysis of patients. It defines the anatomical extent of lung cancer, provides the criteria to distinguish specific patients, and makes the clinical cohort studies easier. Stage groups are defined by the specific primary tumor (T), nodal status for metastasis (N), and metastasis at the distant organs (M).

Nodal involvement is a critical factor that could predict the prognosis after surgery [7, 8], but there is still no accurate evidence to illustrate the influence of the extent of LN involvement in NSCLC. Although the pathological lymph nodes stage has long been a basic criterion of TNM staging system, it could not evaluate the prognosis of patients more accurately. Particularly, for mediastinal LN involvement, the prognostic values were not well stated. Thus, the border of N classification was difficult to define due to its ambiguity and complexity. And the TNM staging system needs to be better evaluated and justified.

The number of PLNs has been proven to have significant influence on certain cancers such as gastric, breast, colorectal, and bladder cancer [9–11]. Moreover, nN had been involved in the classification of these cancers. Studies also showed that nN was an essential prognostic factor for resected NSCLC [12–15]. Herr et al. [12] showed that nN staging was a better prognostic indicator than pN staging. Lee et al. [16] also demonstrated that the number of PLNs was an important prognostic factor for resected NSCLC, consistent with the result of study by Fukui et al. [17]. Similarly, a previous study led by David et al. also demonstrated that the number of LNs sampled (NLNS) influenced both OS and cancer-specific survival (CSS) for NSCLC patients [18]. And the authors concluded that NLNS was a predictor of OS and CSS for NSCLC. All of the above studies have demonstrated that the number of lymph nodes could be used as a predictive and prognostic indicator among NSCLC patients. The N stage classification in the latest 8^th^ edition had similar content as the previous 7^th^ edition, with only the addition of the subgroup of pathology position, which defined N1 to N1a and N1b, N2 to N2a1, N2a2, and N2b by stops of LN involved. However, whether the number of PLNs might be a more accurate disease indicator in NSCLC had not been illustrated. To date, determining the best prognostic index of lung cancer for nN stage or pN stage is not yet clear.

According to the staging manual of the Thoracic Oncology of the International Association for the Study of Lung Cancer, at least six LNs/stations should be histologically confirmed to be nonmetastatic and subsequently can be defined as pN0, but it did not mention the exact number of resected LN to predict the prognosis of NSCLC [3]. Saji et al. suggested ten as a cutoff value [14]. Other studies [19, 20] showed that 16 examined LNs could better evaluate the disease staging and postoperative care. According to the previous studies, dissected LN number ranges from 10 to 18. Regarding the calculation of the number of metastatic LNs, optimal surgery should initially be performed. It had been confirmed that there was no significant difference in survival between selective LN dissection and complete LN dissection, but controversy still exists as regards this finding.

Patients had shorter operative time, lesser blood loss, and fewer morbidity rates in selective LN dissection. Therefore, fewer LN resection was recommended to reduce operative risks and to achieve a better postoperative recovery. In this case, we selected patients with at least 10 resected LNs as a criterion in the study. Another critical point in this study was as follows: proper ways to accurately classify the number of metastatic LNs. Among the previous studies, there were some subgroups of nN classification, but they were just divided randomly or empirically. In our study, we reasonably classified the number of metastatic LNs into three subgroups using the X-tile model. It accurately determined the category of nN, which made the further study more convincing.

The nN stage was well agreed with the pN stage, which were both favorable prognostic factors of OS. However, the definition of the pN stage was too extensive and inaccurately sufficient to evaluate the tumor progression. Thus, the number of metastatic LNs might provide significant information to the existing TNM staging system and could be a complement to the pN stage. Moreover, the survival curves of hypothetical nN stage in Figure 2(b) have also been confirmed in our previous study [21]. Although there were significant differences in the enrolled patients, the survival curves of nN stage were similar, suggesting that it was feasible to incorporate the number of lymph nodes into the current TNM stage. Since the primary tumor's characteristic was an essential factor to determine treatment strategy and prognostic care, we also compared the OS in each pT stage based on the nN classification. As expected, whatever the pT category was, nN classification exhibited an excellent prognostic predictive value. It indicated that nN was an excellent parameter that could be considered as a classification factor.

Thus, we provided a novel classification that combined pN stage with the nN stage for nodal involvement assessment. In conclusion, the subcategory of the OS was significantly distinguished. When we divided pN1 into two subgroups by nN, the OS decreased with higher PLN number. Furthermore, a similar observation was found with pN2 stage and the subgroups. One group [14] also performed similar analysis, but no exact conclusion was demonstrated. They did not show a significant difference in pN0 and pN1 + nN1-3 stages in their studies, but in this study, a significant difference was observed. Survival differences were confusing on the two nN4- groups. However, when we divided nN4- into two subgroups, pN1 + nN4- and pN2 + nN4-, the survival clearly decreased with more anatomical metastasis. Our study has provided substantial evidence that the new classification had better prognostic indication than the old TNM staging system.

Interestingly, the survival was higher in pN2 + nN1-3 compared to that of pN1 + nN4- within the first 5 years. It indicated that the location of metastatic LN played a prominent role for the prognosis in the early time. Hence, proper postoperative treatment and care could be better applied with different groups to have a longer survival time. Some studies showed that both the location and disease burden could affect the prognosis, but the differences were unknown. In this study, we demonstrated that a combination of pN and nN significantly distinguished the classification, particularly the cutting edge of pN1 and pN2.

However, there were still some limitations when considering nN stage as a classification criterion. It is more like a pathologically proof for prognosis rather than a determinant factor for proper treatment. Thus, an accurate method that could identify all the PLNs is significantly required. Although various methods could be used to evaluate the malignancy, such as magnetic resonance imaging, positron emission tomography scan, fine needle aspiration biopsy, and specific tumor markers, none of them were sufficiently reliable. Hence, with the advancement of modern technology, better methods are significantly required. Since it is difficult to include the number of all PLNs, we need to combine the pN stage with the nN stage to evaluate the OS. Data from the SEER database are mainly based on White patients; thus, investigation regarding cancer staging system on other racial groups is also significantly important. Therefore, further study is required to evaluate if nN should be involved in the TNM staging system.

## 5. Conclusion

In our study, we found that the nN stage was a significant prognostic factor on OS for NSCLC patients. Furthermore, the combination of the anatomical location and the number of LNs involvement in NSCLC patients had a better prognostic value than the current TNM staging system based on only pN stage. These results also need further studies to confirm the validity of the novel TNM staging system.

## Figures and Tables

**Table 1:** Non-small cell lung cancer (NSCLC) patient characteristics from SEER database.

Variables	Number	%
Age		
<65	6158	38.9
≥65	9662	61.1

Race		
White	13483	85.2
Black	1244	7.8
Others	1093	6.9

Sex		
Female	7513	47.5
Male	8307	52.5

Histology		
Adenocarcinoma	9587	60.6
Squamous	6233	39.4

T stage		
T1	5563	35.2
T2	7910	50.0
T3	1047	6.6
T4	1300	8.2

pN stage		
pN0	10039	63.5
pN1	3227	20.4
pN2	2505	15.8
pN3	49	0.3

nN stage		
nN0	10304	65.1
nN1–3	3669	23.2
nN4-	1847	11.7

**Table 2:** Influence of different variables on overall survival (OS) by pN stage for patients with NSCLC analyzed by Cox proportional hazard model.

Variables	Univariate analysis		Multivariate analysis	
	Wald *χ*^2^	*P*	HR (95% CI)	*P*
Age	286.45	<0.001		<0.001
< 65			Reference	
≥ 65			1.578 (1.497–1.664)	<0.001

Race	18.08	<0.001		<0.001
White			Reference	
Black			1.019 (0.927–1.121)	0.691
Others			0.799 (0.719–0.887)	<0.001

Sex	134.46	<0.001		<0.001
Female			Reference	
Male			1.359 (1.290–1.431)	<0.001

Histology	48.72	<0.001		<0.001
Squamous			Reference	
Adenocarcinoma			0.831 (0.789–0.875)	<0.001

pT stage	273.10	<0.001		<0.001
pT1			Reference	
pT2			1.345 (1.267–1.429)	<0.001
pT3			1.951 (1.770–2.151)	<0.001
pT4			1.837 (1.679–2.011)	<0.001

pN stage	633.92	<0.001		<0.001
pN0			Reference	
pN1			1.630 (1.534–1.733)	<0.001
pN2			2.157 (2.023–2.300)	<0.001
pN3			2.799 (1.983–3.949)	<0.001

**Table 3:** Influence of different variables on overall survival (OS) for patients by nN stage with NSCLC analyzed by Cox proportional hazard model.

Variables	Univariate analysis		Multivariate analysis	
	Wald *χ*^2^	*P*	HR (95% CI)	*P*
Age	269.89	<0.001		<0.001
< 65			Reference	
≥ 65			1.555 (1.475–1.639)	<0.001

Race	17.84	<0.001		<0.001
White			Reference	
Black			1.022 (0.930–1.124)	0.649
Others			0.801 (0.721–0.889)	<0.001

Sex	137.28	<0.001		<0.001
Female			Reference	
Male			1.363 (1.294–1.435)	<0.001

Histology	49.25	<0.001		<0.001
Squamous			Reference	
Adenocarcinoma			0.831 (0.789–0.875)	<0.001

pT stage	286.01	<0.001		<0.001
pT1			Reference	
pT2			1.340 (1.261–1.422)	<0.001
pT3			1.967 (1.785–2.169)	<0.001
pT4			1.870 (1.709–2.046)	<0.001

nN stage	696.79	<0.001		<0.001
nN0			Reference	
nN1–3			1.657 (1.564–1.756)	<0.001
nN4-			2.371 (2.213–2.540)	<0.001

## Data Availability

The datasets used and analyzed during the current study are available from the corresponding author on reasonable request.

## Abbreviations

NSCLC: Non-small cell lung cancer
TNM: Tumor-node-metastasis
LNs: Lymph nodes
PLNs: Positive lymph nodes
SEER: Surveillance epidemiology and end results
HR: Hazard ratio
CI: Confidence interval
OS: Overall survival
pN: Pathological LNs
nN: Number of LNs
CSS: Cancer-specific survival
NLNS: The number of lymph nodes sampled.
